# P-2070. SARS-CoV-2 Vaccination Rates Among a Cohort of Pediatric Solid Organ Transplant Recipients

**DOI:** 10.1093/ofid/ofae631.2226

**Published:** 2025-01-29

**Authors:** Christopher Reis, Elizabeth H Ristagno, Theresa Madigan

**Affiliations:** Mayo Clinic, Rochester, Minnesota; Mayo Clinic, Rochester, Minnesota; Mayo Clinic, Rochester, Minnesota

## Abstract

**Background:**

SARS-CoV-2 vaccination has been shown to prevent severe disease and subsequent secondary infections in immunocompromised patients. This study assessed the rate of SARS-CoV-2 vaccinations among pediatric solid organ transplant (SOT) recipients at a single transplant center.

**Figure 1.**
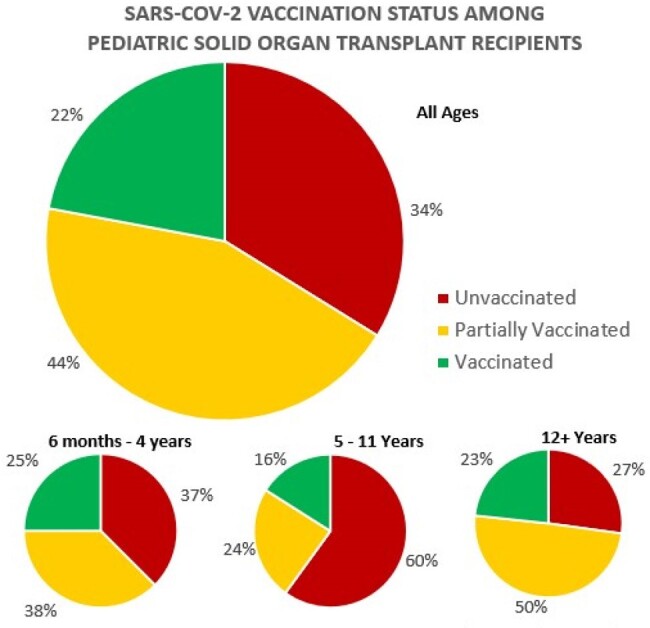
SARS-CoV-2 vaccination status among pediatric solid organ transplant recipients for all ages and by age group.

**Methods:**

We retrospectively reviewed medical records of pediatric recipients of heart, kidney, and liver transplants at Mayo Clinic, Rochester, MN between January 2011 and December 2023. All SARS-CoV-2 vaccine doses administered on or before April 1, 2024 were recorded. Up-to-date (UTD) status was based on current CDC recommendations for immunocompromised patients for each age group. Deceased patients, those no longer following at Mayo Clinic and whose vaccine records were unavailable were excluded. We also assessed whether patients had been seen by an ID physician in the year prior to collection stop date.

**Results:**

The study included 136 patients: 48 kidney, 58 heart and 30 liver transplant recipients; 52.9% were male. Two patients subsequently received organs of a different type. Mean age was 16.1 years. Since authorization in 2020, 90 patients (66.2%) received at least one SARS-CoV-2 vaccine. This varied by age group, with 72.8% of > =12 year olds, 40.0% of 5-11 year olds and 62.5% of under 5 year olds (p = 0.007 [Figure 1]). Seventy-three patients (53.7%) completed at least three doses. Based on current recommendations that include the 2023-2024 formulation, 30 patients (22.0%) were UTD. This did not differ between age groups (p = 0.717) or by transplanted organ type (p = 0.21). Patients that saw ID were significantly more likely to be UTD (p = 0.001). Among partially immunized patients, 95% (n = 57) needed one additional dose to be UTD.

**Conclusion:**

Despite simplified guidelines with fewer doses needed, a third of pediatric SOT recipients remain unvaccinated against SARS-CoV-2 and only 22% are UTD based on current recommendations. The proportion of unvaccinated patients was highest in the 5-11 year old age group and lowest in the oldest. Those that recently saw ID were significantly more likely to be UTD. Our data suggest that continued care provided by ID physicians may increase vaccine uptake, but reasons for undervaccination and strategies to counteract this should be further explored within this vulnerable population.

**Disclosures:**

All Authors: No reported disclosures

